# The Quest for the Holy Grail of Glaucoma Surgery: Does Cypass Herald the End?

**DOI:** 10.5005/jp-journals-10028-1252

**Published:** 2018

**Authors:** Shibal Bhartiya, Tarek Shaarawy

**Affiliations:** 1 Glaucoma Services, Fortis Memorial Hospital, Gurugram, Haryana, India; 2 University of Geneva, Glaucoma Sector, University of Geneva Hospitals, Switzerland

## Abstract

**How to cite this article:** Bhartiya S, Shaarawy T. The Quest for the Holy Grail of Glaucoma Surgery: Does Cypass Herald the End?. J Curr Glaucoma Pract 2018;12(3):99-101.

The advent of minimally invasive glaucoma surgery (MIGS) fractioned the world of glaucoma practice into two distinct groups: those who believed, and those who did not. Minimally invasive glaucoma surgery, a term at best poorly defined, was touted as a panacea for all the evils that plagued the glaucoma surgeon, be it poor compliance to medication, the ephemeral nature of SLT, or the dreaded complications of conventional glaucoma surgeries. The naysayers were quick to coin the moniker, “minimally effective glaucoma surgery (MEGS)” pointing out its potentially lesser efficacy as compared to conventional surgeries. Those on the MIGS bandwagon credited it to observer envy, and indeed, as the focus of innovations in glaucoma surgery, glaucoma surgery shifted from efficacy and cost effectiveness, to safety and efficacy, MIGS seemed here to stay.^1,2^

Regardless of what your position is on the MIGS debate, the recent news of Alcon's voluntary and abrupt global market withdrawal of its CyPass Micro-Stent has brought into sharp focus what has always remained in the periphery of the glaucoma surgeons’ field of vision: the noxious effect of glaucoma surgery on corneal endothelium.^3^

## ENDOTHELIAL CELLS, OR WHAT WE CHOOSE TO IGNORE

Glaucoma, *per se* as well as its management, may have deleterious effects on the corneal endothelium. The possible mediators of this damage may include increased IOP, mechanical forces (especially in the case of aqueous shunts and implantable MIGS), inflammation (both subclinical and otherwise) and the aqueous environment.^4^

Even though there is a paucity of high-quality data, there is enough evidence to suggest that the endothelial cell loss (ECL) in glaucoma patients is more than in those without. In fact, after 1 year of medical treatment of glaucoma, the mean percent loss in endothelial cell density from baseline has been reported to be 3.6%, 4.5%, and 4.2% for the dorzolamide, timolol, and betaxolol groups, respectively.^5^

Also, we know that ECL after glaucoma surgery is inevitable and usually significant: be it trabeculectomy, conventional aqueous shunts, ExPRESS shunt, or even laser iridotomy.^6,7^ In fact, despite the relative safety of laser peripheral iridotomy, the eventual development of focal or generalized corneal decompensation has been described by several authors. This ECL usually consists of mild to moderate endothelial loss on specular microscopy, but we have all encountered, albeit rarely, corneal decompensation following glaucoma surgery.^6–18^

Following SLT, transient corneal endothelial abnormalities have been reported, and long-term effects are presumed to be negligible in normal corneas or single treatments. However, authors recommend caution in compromised corneas and corneas with pigment deposits on the endothelium. These patients may be at a higher risk of further corneal endothelial compromise, especially after repeated SLT.^8,9^

ECL following conventional glaucoma surgery has been reported to range from 2.5 to 18.6%, over a variable period of 3 to 24 months. Authors have reported an ECL following trabeculectomy ranging from 2.5 to 14.5% over three months.^10–14^ The ECL following deep sclerectomy at 24 months has been reported to be 2.6%.^10^

The conventional aqueous shunts have, predictably, not fared much better. The ECL following the Ahmed glaucoma valve has been reported to range from 6.9% at 6 months, to 12.3–18.3% at 24 months.^14–17^ Molteno implantation has been reported to result in an ECL of 12.4% at 24 months,^16^ while the same following Baerveldt implantation has been reported to be 13.6% at 36 months.^17^

As for MIGS, there were also no significant changes in endothelial cell density over one year following the XEN gel stent implantation, both with and without cataract surgery; however, the authors did report a significant increase in cell hexagonality.^18^

The change in endothelial parameters after Hydrus implantation along with phacoemulsification was comparable to the ones of patients who underwent cataract surgery alone, at six months of follow up.^19^ The Hydrus Microstent was approved by the USFDA in August 2018, based on the two year data of the HORIZON study. Ivantis released the 3-year follow-up data recently, in the wake of the ECL related CyPass withdrawal. They reported that the mean central cell counts for both treatment and cataract surgery groups are stable, showing a 1% loss through two years compared to post-operative, and an additional 1% loss from year 2 to year 3. In addition, the data also reveals that the between-group difference in the percentage of patients with 30% ECL was stable between the 1 year and 2 year follow-up, and declined slightly at the 3 year follow up. They also reported no correlations between ECL and the position of the Hydrus, or any device migration.^20^

## SO WHAT EXACTLY IS THE CYPASS ECL CONTROVERSY?

In the five year post-approval extension of the COMPASS trial, called the COMPASS-XT, patients who had undergone phacoemulsification combined with a CyPass Micro-Stent, were found to have a significantly greater reduction in endothelial cell counts than patients who had phacoemulsification alone. At the time of publication, details of the COMPASS-XT are sketchy, but the definitive reason for withdrawal of the CyPass Microstent has been cited as a greater ECL at five years, than reported at the end of two years in the COMPASS trail. At 36 months, there were only 36 patients of the original 200 cases, and 53 controls were available for review, therefore precluding any meaningful interpretation of complications. An ECL of 30% at 5 years is considered to be clinically significant as per the ANSI Z80:27 standards, and this was seen in 27.2% patients with CyPass, as against 10% in the controls. That said, apart from the ECL, the report does not present any other significant safety concerns.^21^

In addition, the report may also suggest a modification in the surgical technique of CyPass Microstent implantation, taking care that no rings are visible on anterior chamber angle gonioscopy or photos, which have been used to grade implantation depth in the COMPASS XT.^3,21,22^

The official statement emphasizes that stents with greater anterior chamber exposure may have greater ECL at 5 years, but does not recommend any intervention in these patients until there are signs of corneal decompensation. The early migration of the Microstent, however, remains a critical variable that requires long term monitoring.^22^

While the financial, legal and industry implications of this decision which has variably been applauded and criticized by those who use MIGS, or do not; has certainly explained how MIGS (and indeed, the Cypass Microstent) is positioned in the glaucoma surgeons armamentarium.

The choice of a particular therapy for the individual patient must be customized, on the basis of the risk-benefit ratio for the particular subject. Indeed, for the early glaucoma patient, the risk of therapy must be very low, as the risk of loss of vision significant enough to impact quality of life, at least in the short-term, is not high. On the other hand, for patients with advanced disease, one can take more risks and choose a surgery known to have a better IOP lowering profile.

This is why comparing the complication rates of conventional glaucoma surgeries (with respect to endothelial cell loss, or otherwise), would be incorrect, since the patient profiles they are chosen for is remarkably different.

Similarly, all of the MIGS may not also be grouped together when looking at their safety profiles and complication rates. Given that Schlemm canal–based or supraciliary-based MIGS procedures, like the Cypass Microstent, are presumed to be less efficacious and believed to cater to a mostly low-risk glaucoma population with mild-to-moderate disease, their required safety standards are set to be higher than that of subconjunctival MIGS. The aim of the MIGS in this case is to primarily obviate the issues of compliance, ocular surface disease or drop load, in patients already scheduled for cataract surgery. In fact, its use is restricted to adult patients with mild-to-moderate open-angle glaucoma in conjunction with cataract surgery, a labeling which may well be up for revision as data about its long-term safety and efficacy becomes available.

The subconjunctival MIGS, on the other hand, are presumed to be more efficacious, and may be used in refractory glaucomas, therefore, the safety standards would understandably be less stringent. Even though there are no head to head trials for now between the subconjunctival and Schlemm Canal-based MIGS, regulatory authorities and surgeons alike have worked with this generic premise.

Which is why the CyPass Micro-Stent gained FDA approval through Premarket Approval (PMA), the most stringent regulatory category for medical devices, while the XEN implant was judged to be “substantially equivalent” to a predicate device, the aqueous shunt implant, and was eligible for FDA registration through the less stringent 510(k) clearance.^3^

The American Society of cataract and refractive surgery (ASCRS) report indicates that ECL is related more to the surgical implantation technique and that a more posterior positioning of the CyPass Micro-Stent would decrease the incidence of endothelial cell loss. A further recommendation was to avoid the use of the device in phakic eyes with angle closure, since a posterior implantation may result in occlusion of the device with iris tissue (www.ascrs.org/CyPass_statement).

The critical learning from the CyPass, for industry and surgeons alike, is the need for continuous vigilance for any approved device, where the excitement of having a new surgical option must be balanced with judicious caution. In addition, peer pressure to jump on the MIGS bandwagon, may just also be the cause of a cognitive bias in the evaluation of these surgeries.

As for MIGS, the regulatory authorities and surgeons will both hopefully continue the post-market surveillance, ensuring safety of all the devices available now, and in the future. In fact, this just may be how all glaucoma surgeries are evaluated in the future, even those hitherto considered the gold standard for glaucoma care. This just might bring the focus of glaucoma surgery back to quality of life of the patient, its safety profile and risk-benefit ratio.

Given the natural history of ECL in glaucoma, patients undergoing cataract surgery may not be the best control group for ECL following MIGS. A more rational and convincing argument about safety of MIGs may be derived from a head to head comparison of the ECL following MIGS, and other conventional glaucoma drainage procedures.

Despite this hiccup in the trajectory of surgical innovations in glaucoma, many are of the opinion that minimally invasive glaucoma surgeries is a safer alternative to conventional glaucoma surgery, and remain an intelligent treatment option for the well-chosen candidate.

The current sequence of events have strongly reiterated the need for customization of glaucoma therapy to the needs and aspirations of the individual patient. The appropriate surgical management, tailored to the individual, considering the risk-benefit ratio of each device/technique is essential before any therapeutic decision may be considered. Key considerations for this choice in current glaucoma practice are severity of disease, compliance, safety and efficacy of the procedure, its learning curve and cost effectiveness, as well as the surgeons’ experience. Keeping this in mind, it would be imprudent to dismiss MIGS, and indeed the Cypass Microstent, as a misadventure. The latter may still prove to be safe in a specific target population, with a modification of surgical technique, in the years to come.

Minimally Invasive Glaucoma Surgery, on the other hand, is definitely here to stay, though still in evolution, in terms of technique and patient choice. Alliterations of currently available devices and newer variants of the same will all shape how we treat glaucoma in the future. Industry, regulators, insurance agencies, glaucoma practitioners and patients together will drive this change, and revolutionize the management of this chronic disease. The quest for the Holy Grail of glaucoma therapy may have suffered a minor setback, but in no way shall it be thwarted.
